# Short Communications: Short technical description of the MonA and PotLab colorimeters

**DOI:** 10.1155/S1463924679000614

**Published:** 1979

**Authors:** S. N. Pocock, J. M. Rideout

**Affiliations:** Divisions of Bioengineering and Clinical Chemistry Clinical Research Centre Watford Road Harrow, Middlesex HA1 3UJ UK


### Short Communications

### Short technical description of the MonA and PotLab colorimeters

By S.N. Pocock and J.M. Rideout
*Divisions of Bioengineering and Clinical Chemistry, Clinical Research Centre, Watford Road, Harrow, Middlesex HA1 3UJ, UK.*

The MonA and PotLab instruments represent the upper and lower limits of a range of rugged colorimeters designed for use in developing countries based on the concept of using a light emitting diode (LED) as an alternative to the tungsten lamp and filter of the conventional colorimeter. The green LED with a maximum emission around 565 nm and a bandwidth of 30 nm was chosen because it is the only colour applicable to a useful range of clinical chemical tests.

Initial testing of LEDs revealed that the light output varies with time until thermal equilibrium is achieved. This problem, coupled with the requirement to keep battery consumption as low as possible, was solved by pulsed operation of the LED.

### Flash operation colorimeter

**MonA**

Light from a single LED, pulsed for a nominal 14 msec, reaches two silicon photodiodes which are connected to the transconductance amplifiers. One photocell 'sees' the LED directly (reference) and the other via the cuvette containing the sample to be measured. The 'zero' control sets the two output voltages to be equal when the sample is reckoned as 100% T, i.e. as reagent blank.

The two amplifiers are connected to what are effectively two sample and hold circuits except that one (the reference) has a controlled exponential droop rate. For the first 10 msec (nominal) of the 'pulsed' time ($t_0$ to $t_1$) these circuits are in the 'sample' mode and acquire their respective amplifier voltages. The 'sample' gates are opened at time $t_1$, and the 'reference' side decays exponentially with the pre-set time-constant. At some point in time ($t_2$) the 'reference' voltage reaches the same level as the 'sample' voltage and this causes a comparator to change its output (hi-to-lo).

The time period from $t_1$ to $t_2$ is a measure, to some scale, of the log of the ratio of the two signal voltages which correspond to the 'reference' and 'sample' light levels seen by the two photocells. The function of the digital circuitry is to measure and display this time which is shown as the number of clock pulses in this interval. Using a simple series of logic gates, an accurately controlled clock and a digital counter, the result is finally displayed on a conventional 3-digit segment LED display. This logarithmic circuit provides good accuracy with economy in components and is very stable to ambient temperature changes up to modest values of absorbance.

While the energising LED is 'on' the display is held 'off', thus maintaining the current drain approximately constant, as each takes about 40 mA from the supply. The LED pulse time is made longer than the acquisition time of the sample-and-hold circuits to eliminate interference due to the switch-off of the flash.

The sequence commences immediately the circuit is powered by pressing the 'READ' button, and repeats at 1 sec (nominal) intervals until the button is released. The 'counting' only occupies a small proportion of 1 sec, so that the display appears 'fixed' to the operator, updating at 1 sec intervals. A simple timer/processor provides the 10 and 14 msec pulses which control the logic, pulse the LED, reset the counter, and the updating.

By suitable selection of the time constant and clock frequency, any appropriate scaling factor can be set. Instrument calibration at two points on the scale is provided by selecting, with push-buttons, a precision resistor attenuator at the output of the 'test' amplifier – an electrical equivalent of optical absorbance.

The electronic circuits operate with ± 3 volt lines derived by a series stabilizer from a single 9 volt battery which can decay to 6.5 V minimum. If each reading occupies only a few seconds, a few thousand readings are possible – a probable battery life of some months under typical conditions.

No power is consumed at other times and this mode of operation, without any warming-up period, ensures minimum battery consumption and obviates leaving the battery 'on' accidentally.

**PotLab**

PotLab represents an attempt to produce a minimum cost haemoglobinometer for use with the haemoglobincyanide method recommended by the International Committee for Standardization in Haematology (ICSH).

The green LED is retained as the light source but it is operated in the continuous mode at somewhat reduced current, about 20 mA. Coupled with the more sensitive photo-detectors, adequate sensitivity is obtained with low power consumption.

Hermetically sealed cadmium sulphide light-dependent resistors (CdS) are used. These devices are more sensitive and somewhat cheaper than silicon photodiodes and can operate with simpler circuitry. At constant voltage the CdS cell produces a current which is a function of illumination with a

$$\text{'gamma'} = \frac{\text{log current change}}{\text{log illumination change}}$$

which is usually about 0.8 to 0.9 but which unfortunately is variable from sample to sample. The cells are also more temperature sensitive than silicon detectors. Their peak spectral sensitivity is advantageously near to the emission of the green LED.

While a direct reading instrument is preferable, the cost of using either a rugged sensitive tropicalised meter-indicator or a digital display is relatively high. A null indicator offers the cheapest solution and in the PotLab a pair of LEDs is used to indicate the direction in which a calibrated potentiometer control must be rotated to achieve balance.

As the name suggests, PotLab uses a potentiometer method of measurement based on two light dependent resistors in a bridge circuit. A section of a quad amplifier integrated circuit provides the drive to the d.c. null indicators and readings are taken from the calibrated potentiometer (scaled in haemoglobin, g/100 ml). This, with fixed resistors, and the two light-dependent resistors (test and reference), comprises the bridge network. The output of the bridge feeds the comparator which drives a 'long tailed pair' of transistors, so illuminating one or other LED null indicator. The remaining section of the quad amplifier is concerned with control of the current through the LED.

A ten-turn potentiometer (set calibration control) is in series with the calibrated potentiometer and is intended to be in mid position for a cell of average gamma with the standard calibration scale. The present potentiometer will take up moderate gamma changes from cell to cell but the degree of error has not been assessed.

The circuit functions with supply voltages from 5 to 18

volts, normal operation being from a 9 V internal battery with the same economical 'press to read' power 'on' button. The effect of non-linearity of the calibration scale is minimised by its generous length, over 255 mm, and the expansion of the scale divisions in the more critical lower range. Zero adjustment is by mechanical microadjustment which moves the reference cell from the side of the LED.

REFERENCE
[1] Hjelm, M., Brown S.S. and Mitchell, F.L. *The Journal of Automatic Chemistry* (1979), 1, 214.

### Report of WHO-sponsored trial of MonA and PotLab colorimeters

**By J.M. Rideout,**
*Division of Clinical Chemsitry, Clinical Research Centre, Harrow, Middlesex HA1 3UJ, UK.*

### Background

The objects of the trial were to field-test, in a variety of climatic conditions, two designs of solid state colorimeter calibrated directly in grams per dl haemoglobin and using a haemoglobinocyanide method for total haemoglobin in blood (based on the recommendations of the International Committee for Standardisation in Haematology) [1] and the Swizzlestick technique [2].

It was originally intended that the trial would take place in two phases. Three digital MonA (Mark 1) colorimeters and three PotLab colorimeters, with the necessary reagents, standards, controls and protocols would be distributed by WHO, Geneva; two to laboratories in South East Asia, two to the Western Pacific and two to the Eastern Mediterranean.

The recipients of the PotLab colorimeters were to carry out haemoglobin estimations only; the recipients of the MonA colorimeters were to carry out total protein and albumin estimations in addition to haemoglobin. When these evaluations had been carried out and the exercises completed, the instruments were to be returned to the Clinical Research Centre (CRC) for checking. The exercises would then be repeated, but the laboratories which originally had a MonA colorimeter would receive a PotLab and vice versa.

In fact the trial was terminated at the end of the first phase partly because the time scale for the exercise became protracted but primarily because sufficient information was forthcoming to make the second phase unnecessary.

In addition to the WHO-sponsored trials, Medical Research Council staff have carried out field trials of the MonA colorimeter system on five occasions in The Gambia and once in Nepal. Some aspects of the experiences gained in these trials have been combined with the WHO reports to produce this assessment.

Both colorimeters use a green light-emitting diode as the light source, thus obviating the need for a conventional filter. PotLab represents an attempt to design the cheapest possible haemoglobinometer. MonA represents a more sophisticated instrument. The observed shortcomings of each of the instruments are as follows:

### PotLab

(1) The 'balance' indicators, small red light-emitting diodes (LEDs) are difficult to see in conditions of high illumination.
(2) Balance is difficult to achieve. As the point of balance is indicated when one LED goes out and the other comes on, it is easy to 'overshoot'. There is a tendency for operators to spend a lot of time trying to produce a 'no lights on' condition.
(3) The mode of operation of the light source (LED) makes it susceptible to thermal drift.
(4) A combination of (2) and (3) produces non-reproducibility of results.
(5) The instrument needs carefully matched cuvettes.

### MonA

(1) The LED numerical display is difficult to see in conditions of high levels of illumination.
(2) The instrument needs carefully matched cuvettes.
(3) In conditons of high humidity the instrument did not indicate the correct value when the 'high calibrate' button was pressed. With one exception, calibration had returned to normal by the time the colorimeter was checked at the CRC.
(4) In one colorimeter, part of the optical section had been damaged by fungal attack.

All MonA colorimeters returned to the CRC have been fitted with an improved optical section which is less susceptible to fungal attack and less sensitive to cuvette imperfections. Two modified colorimeters are presently undergoing field trials.

The circuit boards of the returned colorimeters have been protected with a silicone rubber coating in an effort to reduce the effects of high humidity. This treatment has only been partly successful and work is in progress to identify the part or parts of the instrument sensitive to humidity.

### General assessment of the usefulness of the system

A colorimeter, a box of cuvettes and the necessary packing material will fit into a cardboard box 21 x 20 x 13 cm; with reagents, etc., the whole can be packed into a box 27 x 33 x 29 cm. Colorimeter systems have been sent by air mail to various parts of the world without suffering damage, and can be used immediately.

The portability of the system has been further demonstrated by the fact that haemoglobin estimations have been performed in native schools and villages, in the absence of a local electricity supply. In addition the accuracy and precision obtained with the MonA colorimeter was previously unobtainable using colour comparison methods.

In many laboratories in developing countries it is impossible to get reproducible readings from colorimeters connected to the local electricity supply because of voltage fluctuations. In these cases the MonA colorimeter has given a significant improvement in precision.

### Conclusions

The WHO/MRC trials have provided a considerable amount of information which indicates that there is a need for two colorimeters with completely different specifications:

(I) A field instrument for use by health care workers in village clinics that can measure haemoglobin in a drop of undiluted blood. A design for a suitable system is being considered.
(II) A general purpose colorimeter for a rural hospital laboratory capable of measuring haemoglobin and at least five other chemical constituents of blood. The instrument, which would be a Mark 2 version of the MonA colorimeter as evaluated in the field trials described above, should incorporate the following features:
(1) It should have two modes of operation, COMPARATIVE and ABSOLUTE.
(a) In the COMPARATIVE mode the following features are required:
(i) A continuously variable scaling factor to allow the

